# Detection of Osteogenic Differentiation by Differential Mineralized Matrix Production in Mesenchymal Stromal Cells by Raman Spectroscopy

**DOI:** 10.1371/journal.pone.0065438

**Published:** 2013-05-29

**Authors:** Pei-San Hung, Yi-Chun Kuo, He-Guei Chen, Hui-Hua Kenny Chiang, Oscar Kuang-Sheng Lee

**Affiliations:** 1 Institute of Biophotonics, National Yang-Ming University, Taipei, Taiwan; 2 Institute of Clinical Medicine, National Yang-Ming University, Taipei, Taiwan; 3 Institute of Biomedical Engineering, National Yang-Ming University, Taipei, Taiwan; 4 Department of Orthopaedics and Traumatology, Taipei Veterans General Hospital, Taipei, Taiwan; 5 Stem cell Research Center, National Yang-Ming University, Taipei, Taiwan; National Cancer Institute, United States of America

## Abstract

Mesenchymal stromal cells (MSCs) hold great potential in skeletal tissue engineering and regenerative medicine. However, conventional methods that are used in molecular biology to evaluate osteogenic differentiation of MSCs require a relatively large amount of cells. Cell lysis and cell fixation are also required and all these steps are time-consuming. Therefore, it is imperative to develop a facile technique which can provide real-time information with high sensitivity and selectivity to detect the osteogenic maturation of MSCs. In this study, we use Raman spectroscopy as a biosensor to monitor the production of mineralized matrices during osteogenic induction of MSCs. In summary, Raman spectroscopy is an excellent biosensor to detect the extent of maturation level during MSCs-osteoblast differentiation with a non-disruptive, real-time and label free manner. We expect that this study will promote further investigation of stem cell research and clinical applications.

## Introduction

Mesenchymal stromal cells (MSCs) are multipotent adult stem cells which were first discovered by Friedenstein's group in bone marrow stoma as fibroblast-like non-hematopoietic stem cells [1] and have the capacity to differentiate into osteocytes, adipocytes, endothelial cells, myocytes, astrocytes, and hepatocytes.[2], [3], [4], [5], [6] MSCs have been isolated from a variety of tissue such bone marrow, umbilical cord blood, [7] adipose tissue, [8] and peripheral blood.[9], [10] Previous studies have shown that MSCs play an important role during bone formation and bone remodeling and also have been indicated as potential resources for many clinical applications. [11], [12], [13], [14]

An osteoblast is bone forming cell which mediates in the bone-homeostasis during osteogenesis [15] and exhibits a typical elongated fibroblast-like morphology with a spindle-like appearance. It has been reported that various extracellular matrices and soluble factors modulated the activity of signaling proteins [16] and transcription factors [17] during MSCs-osteoblast differentiation. Current strategies to induce MSCs osteogenesis include mechanical factors such as matrix stiffness [18], [19] and chemical stimuli such as β-glycerol phosphate, [7] dexamethasone,[20] and ascorbic acid. [21] The mineral matrices which produce by bone cells are widely applied in tissue engineering and regenerative medicine researches for enhancement of osteogenesis or as maturation biomarkers of MSC-osteoblast differentiation. [22], [23], [24], [25], [26]

To evaluate the maturation level of MSCs-osteoblast differentiation of, histochemical and molecular biological methods such as alkaline phosphatase staining, Von Kossa staining, Western blot and reverse transcription polymerase chain reaction (RT-PCR) are commonly used.[27], [28], [29] However, all these traditional methods are time-consuming, cell-destructing, and can only offer semi-quantitative or non-quantitative information except for real-time RT-PCR. Moreover, these conventional methods to detect the extent of osteogenic differentiation are not in situ and require cell lyses or fixation, which causes cell death and makes continuous observation very difficult. A sensitive and objective method for research studies and clinical applications is not still available.

Progress has been achieved to introduce vibrational spectroscopy including Raman and Infrared spectroscopy (IR) into medical diagnostics and cellular biology over the past decade. Most of the clinical diagnostics carry out with infrared microspectroscopy which offers much higher speed and a spatial resolution of about the size of a cell.[30], [31] However, water or liquid solvents cannot be used during IR measurement and sample preparation should be elaborate. To investigate the chemical and biological properties changes during bone formation process in MSCs, Raman spectroscopy has been used in this study due to its high spatial resolution, high sensitive of biological changes at a subcellular level detection and the sample for Raman measurement can be almost in any state. [32], [33]

Raman spectroscopy has been widely used as an in situ and single cell detector for a wide variety of biological applications recently. [34], [35], [36]The Raman spectroscopic technique provides a detail molecular structure, chemical composition and molecular interaction in tissues and cells.[37], [38], [39], [40], [41] The molecular composition and structural changes, concomitant with the disease progression, are often revealed in the spectra. Hence, any set of quantitative spectral changes, sufficiently specific to a particular state of differentiation, can be used as phenotypic markers of the disease.[42] Previous studies have illustrated the differences between Raman spectra of the healthy vs. the diseased cells or tissues, [43], [44] and the feasibility of Raman spectroscopic fingerprinting of cells for clinical diagnostic applications has been successfully demonstrated.[43], [44], [45], [46]

The potential of Raman spectroscopic technique is evaluated to detect the maturation level during MSCs-osteoblast differentiation in live cells through detecting the extent of mineralized matrices which are reported to possibly be involved in osteogenesis including amorphous calcium phosphate (ACP)[47], octacalcium phosphate (OCP)[48], hydroxyapatite (HAP)[49], [50], β-tricalcium phosphate (β-TCP) [51] and dicalcium phosphate dehydrate (DCPD) [51] in this study.

## Materials and Methods

### Culture maintenance and expansion

For studies involving human tissues we obtained Institutional Review Board approval of Taipei Veterans General Hospital with written informed consents. MSCs were isolated from bone marrow collected from healthy young donors during fracture surgery, and purified with negative immuno-selection and limiting dilution as previously described.[7] Expansion medium for MSCs consists of Iscove's Modified Dulbecco's medium (IMDM; Gibco, Grand Island, NY) and 10% ES fetal bovine serum (ES-FBS; Sigma-Aldrich, St Louis, MO), supplemented with 10 ng/ml basic fibroblast growth factor (bFGF; R&D systems, Inc., Minneapolis, MN), 10 ng/ml epidermal growth factor (EGF; R&D system, Inc.), 100 U penicillin, 1000 U streptomycin, and 2 mM L-glutamine (PSG; Gibco).

### Osteogenic differentiation of MSCs

Osteogenic induction was carried out according to previously reported protocols [29]. To induce osteogenic differentiation, MSCs were cultured to the density of approximately 40% confluence before treatment with the osteogenic induction medium consisting of IMDM supplemented with 0.1 µM dexamethasone (Sigma-Aldrich), 10 mM β-glycerol phosphate (Sigma-Aldrich), and 0.2 mM ascorbic acid (ASA; Sigma-Aldrich). MSCs were treated with osteogenic medium for 24 days in which medium was changed every 3 days.

### RNA extraction and real-time polymerase chain reaction (qPCR)

RNA prepared from 3x10^5^ in vitro culture cells and total RNA was isolated using Trizol (Invitrogen) and cleaned using an RNA easy minikit (Quiagen, Courtaboeuf, France). We reverse transcribed the messenger RNA to complementary DNA using reagents (Genemark Technology, Taiwan) according to the manufacturer's instructions. Quantitative real-time PCR analysis of total RNA from cultured cells was performed using ABI Step One Plus Real Time PCR System. cDNA was amplified using an ABI Step One Plus Real Time PCR System at 95°C for 60 seconds, 56°C for 45 seconds, and 72°C for 60 seconds for 40 cycles, after initial denaturation at 95°C for 5 minutes. The primers used for amplification are 5′-gtgcctaggcgcatttca-3′ (forward) and 5′-gctcttcttactgagagtggaagg-3′ (reverse) for runt-related transcription factor 2 (RUNX2); 5′-gaaccaaaaattaaagtgattgaagg-3′ (forward) and 5′-tgacttttgttagtgtgggtcct-3′ (reverse) for periostin, 5′-cccctggaaagaatggagat-3′ (forward) and 5′-aatcctcgagcaccctgag-3′ (reverse) for type I collagen; internal control, 5′-gctggcccatagtgatcttt-3′ (forward) and 5′-tccttgggttatcttcacacg-3′ (reverse) for TATA-binding protein (TBP). The relative expression levels of mRNA in cells were normalized by internal controls, non-differentiated controls and determined with ΔΔCT.

### Cytochemical staining of osteogenic differentiation

For histochemical analysis of osteogenesis, cells were rinsed twice with phosphate buffered saline (PBS), fixed with 3.7% formaldehyde for 20 minutes, and washed with distilled water. Mineralization matrix was analyzed with Von kossa staining using 1% silver nitrate (Sigma-Aldrich) under UV light for 45 minutes, followed by 3% sodium thiosulfate (Sigma-Aldrich) for 5 minutes, and then counterstained with van Gieson (Sigma-Aldrich) for 5 minutes and with Alkaline phosphatase histochemical stain using the BCIP/NBT (5-bromo-4-chloro-3-indolyl phosphate/nitroblue tetrazolium; Sigma-Aldrich) solution in dark for 45 minutes.

### Raman spectroscopy

In this study, a 18-mW He-Ne laser operating at 632.8 nm was used to provide the Raman excitation. An 80-cm focal length spectrometer system (LabRAM HR 800, Jobin Yvon, Longjumeau Cedex, France) was equipped with a BX-41 Olympus confocal optical microscope and a 60X water immersion M-Plan objective (NA = 0.9). A liquid-nitrogen-cooled CCD 2D array detector was used to measure the Raman signal by integrating for 30 second. Spectra were recorded from 800 to 1800 cm^−1^ with a spectral resolution of approximately 5 cm^−1^.

### Specimen preparation

**Figure 1:**
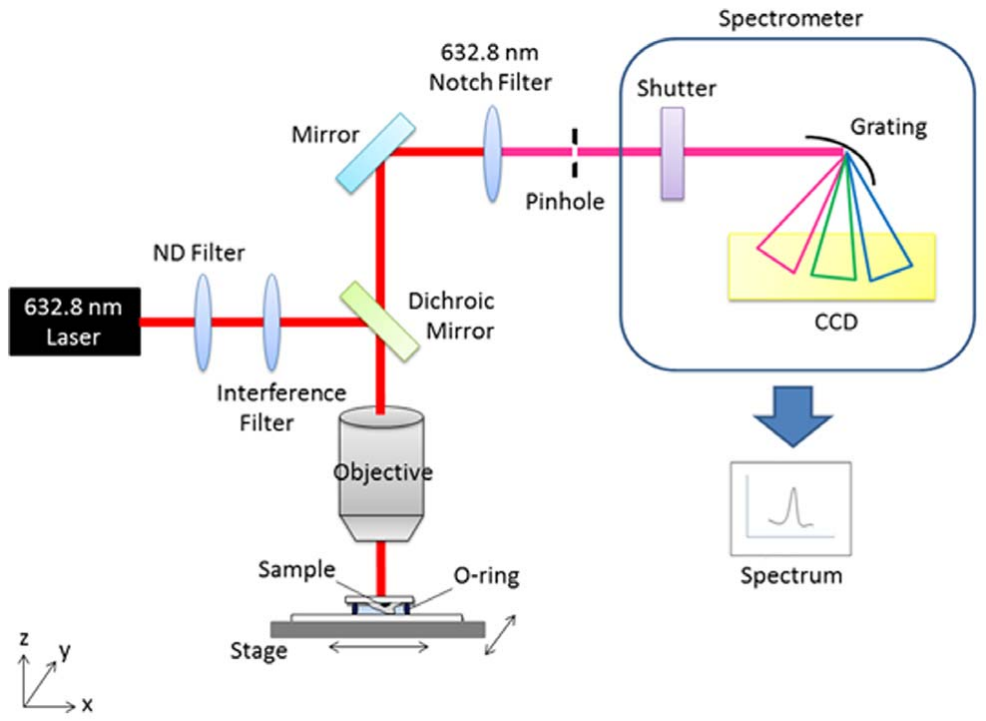
Schematic diagram of the Raman platform set-up.

MSCs seeded on 2 mm×2 mm quartz coverslip and treated osteogenic induction medium. Before measurement, cells were washed with PBS for twice, and the coverslip was placed on quartz slide. An O-ring placed between coverslip and slide. The minimized chamber was filled with PBS to prevent cell death during the measurement, as was shown in Figure 1.

### Data analysis

Each Raman spectrum represents the average of 5 different replicates on one single cell surface. Labspec 5.0 software was used for signal processing. All spectra in this study were normalized to CH_2_ wag at 1449 cm^−1^. A Phe background spectrum at 1004 cm^−1^ was acquired for each individual substrate used, and subsequently subtracted before averaging. [41], [52], [53], [54], [55], [56], [57], [58]

## Results

### Raman signals of background

**Figure 2:**
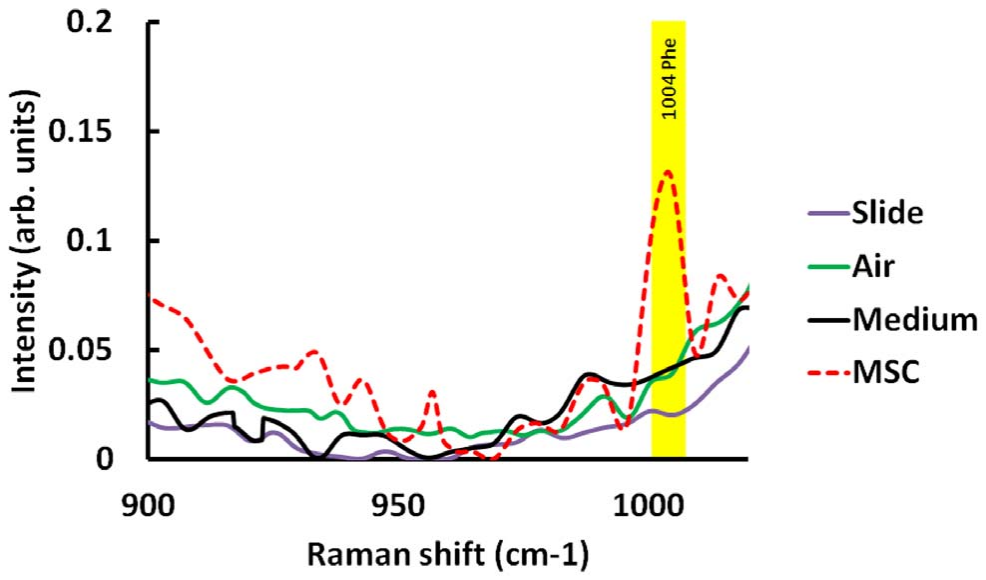
(A) Background signal of Raman spectra. Magnified details of the region for control and cell species from 900 to 1020 cm^−1^ in stack diagram.

The background signals of quartz slide and quartz slide with coverslip have been found at 800 cm^−1^ and 1065 cm^−1^ (Figure S1). To subtract the compounds from the background, we took the Raman spectrum of background signal from quartz slide, quartz slide combined with quartz coverslip in air, and MSC growth medium and MSC with growth medium which were obtained by averaging over 5 different locations for 30 seconds integration time. The Raman signal of cellular component, Phenylalanine (Phe), at 1004 cm^−1^ is significantly higher in MSCs compared to control groups. Our results show that mineralized matrix signals in cells are not interfered with background signals, as was shown in Figure 2. These findings indicated that experimental setup of Raman spectroscopy is a great tool for detecting the mineralized matrix signals specifically in live cells during MSC-osteoblast differentiation.

### Raman spectra of MSCs and MSCs-derived osteoblasts

**Figure 3:**
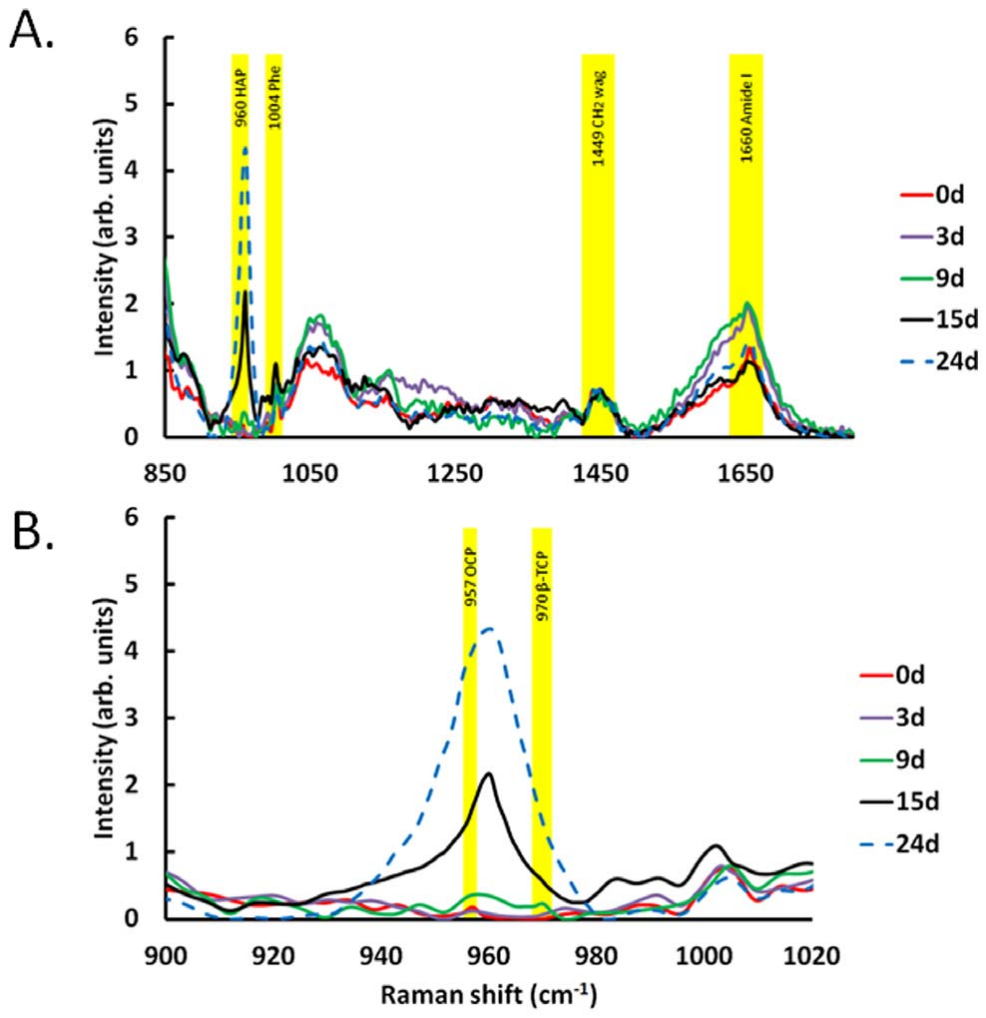
(A) Raman spectra of MSCs during osteogenic differentiation from 900 to 1800 cm^−1^. (B) Magnified detail region for species from 900 to 1020 cm^−1^ in stack diagram. OCP at 957 cm^−1^ decreased upon osteogenic differentiation; β-TCP at 970 cm^−1^ transiently appeared at Day 9 and HAP at 960 cm^−1^ significantly increased after day9.

In order to elucidate the differential production of mineralized matrix during osteogenic differentiation of MSCs, Raman spectra were recorded on day 0, 3, 9, 15 and day 24 for three times, and a consist result which was shown in Figure 3A. The region of Raman spectra of cellular components and mineral species were marked in yellow. Previous studies have demonstrated that OCP is the precursor of mineralized matrix at the early stage mineralization and also involve in HAP synthesis during bone formation.[59], [60] The Raman signal of OCP at 957cm^−1^ shows in MSCs at Day 0 without osteogenic induction and signal intensity decreases from Day 3 after osteogenic induction. The OCP signal cannot be detected after Day 9 osteogenic induction. In addition, a Raman signal of β-TCP at 970 cm^−1^, is a HAP precursor, is found after Day 9 osteogenic induction (Figure 3B).[61] As expected, the intensity of Raman signal of HAP at 960 cm^−1^ slightly increase after Day 9 and highly correlates with induction time from day 9 to Day 24. According to our data, OCP might be dissolved for mineralization. As the result of that OCP signal can be used as precursor marker which shows in non-differentiated MSCs and slightly decreases when cells underwent oesteogenic differentiation. Furthermore, β-TCP contributes to HAP synthesis which can be used as an early stage marker for MSC-osteoblast differentiation so the intensity of β-TCP signal rises at Day 9 under osteogenic induction and quickly disappeared afterward.

The signals of ACP and DCPD are undetectable throughout the experiments. The Raman spectrum in Figure 3A consisting of peaks corresponding to the molecular vibration of various cellular components including of Phe at 1004 cm^−1^, amide I at 1660 cm^−1^, and carbohydrate CH_2_ wag at 1449 cm^−1^ were observed. Under osteogenic induction, the HAP signal increases significantly at 960 cm^−1^. To investigate the expression levels of mineral components in MSCs during osteogenic differentiation, we magnified Fig. 3A. As show in Fig. 3B, Raman spectrum was dissected in detail as depicted in the region from 900 to 1020 cm^−1^. These data shows that OCP at 957 cm^−1^ represent is significantly higher in the undifferentiated MSCs, but decreased quickly after 6th day of osteogenic induction which data was not shown. The OCP signal cannot be found after day 9. On the contrary, we found the β-TCP at 970 cm^−1^ transient appears at the Day 9. The Raman signal of HAP at 960 cm^−1^ dramatically increased from Day 9 under osteogenic induction in MSCs. These results demonstrate that Raman spectroscopy is a powerful tool to measure the maturation level of MSC-osteoblast differentiation by detecting the intensity changes of mineral components in MSCs. However, the ACP at 952 cm^−1^ and DCPD at 985 cm^−1^ cannot be found in Figure 3A.

**Figure 4:**
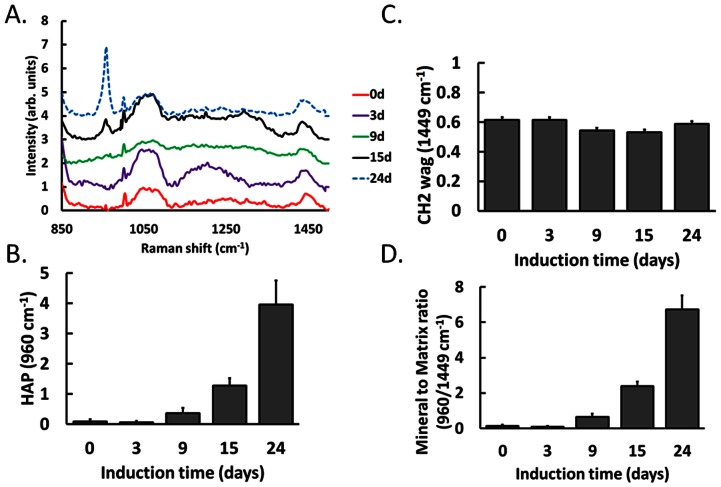
(A)The enhanced Raman spectra of MSCs during osteogenic differentiation from 900 to 1500 cm^−1^. The mineral-to-matrix ratios, HAP/CH_2_ wag (960/1449 cm^−1^), divide the Raman intensity of HAP at 960 cm^−1^(B) and CH_2_ wag 1449 cm^−1^(C). (D) The ratio significantly increased at the later stage of osteogenic induction. Data are shown as mean ± SE (n = 5).

To further confirm the negative results of ACP and DCPD in Figure 3A, we enhanced Raman signals by increasing the integrated duration to two times and reduced the recorded range of Raman shift. The similar results were presented in Fig. 4A, OCP and β-TCP signals transiently appear, and HAP signal increases from Day 9 during MSC-osteoblast differentiation. In addition, ACP and DCPD still cannot be detected in this experiment.

### Quantitative data analysis

It has been reported that mineral-to-matrix ratios (HAP/CH_2_ wag) as a quantitative method to quantify the Raman signals of mineralized matrices. [62], [63] In order to quantitative analyze the experimental results, we calculated the mineral-to-matrix ratios which resulting from dividing the intensity of HAP and CH_2_ wag at 960 cm^−1^ (Figure 4B) and 1449 cm^−1^ (Figure 4C). As expected, Figure 4D suggests that mineral-to-matrix ratios (HAP/CH_2_ wag) increases linearly and highly correlated with the osteogneic induction time and differentiation maturation level during osteogenesis in MSCs.

### In Vitro analysis of MSCs under osteogenic induction

**Figure 5:**
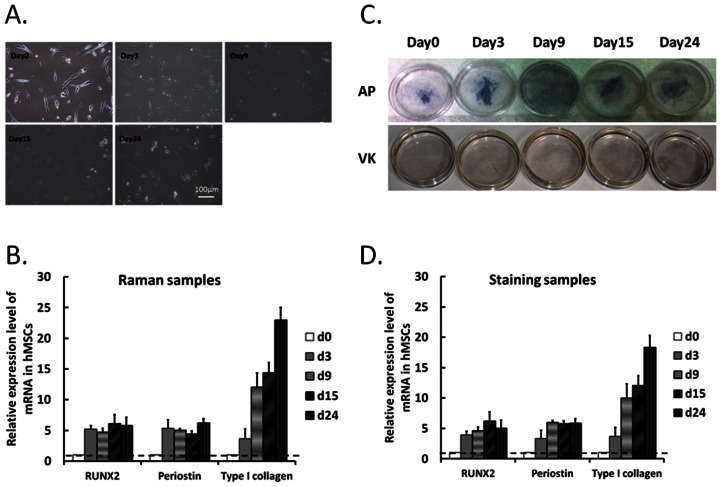
(A) Micrographs of MSCs treated with osteogenic media for 0, 3, 9, 15 and 24 days. (Scale bar: 100 µm) (B) Gene expression profiles of RUNX2, periostin, and type I collagen were detected in MSCs after Raman measurements by qPCR and normalized by internal and undifferentiated controls. Data are shown as mean ± SE (n = 3). (C) Alkaline phosphatase and Von Kossa staining of MSC-osteoblast differentiation. (D) Gene expression profiles of RUNX2, periostin, and type I collagen in staining samples were detected by qPCR and normalized by internal and undifferentiated controls. Data are shown as mean ± SE (n = 3).

To validate the efficiency of osteogenic induction in MSCs, conventional molecular biological techniques were used to evaluate the mineralization level at different time points. Significant changes of cell morphology during MSC-osteoblast differentiation were observed on day 0, 3, 9, 15 and day 24. MSCs underwent morphological changes from spindle-like to flattened cells during osteogenic induction as shown in figure 5A. To confirm the differentiation efficiency in MSCs for Raman measurements, we evaluated the osteogenic lineage markers RUNX2, periostin and type I collagen mRNA levels in MSCs at each time point after Raman measurements. The mRNA expression levels of osteogenic lineage markers up-regulated in MSCs during osteogenic differentiation (Fig. 5B). Mineralized matrixes alkaline phosphatase and calcium phosphatase has been recognized as an early stage and mature bone cell marker that increased with the extent of osteogenic differentiation. The alkaline phosphatase staining and Von Kossa staining were used to evaluate the mineralized matrixes level in MSCs during MSC osteogenesis (Fig. 5C). These data indicated the successful induction of MSCs to differentiate into osteoblasts by our published protocol.[3], [7]And the osteogenic lineage markers RUNX2, periostin and type I collagen mRNA levels of Von Kossa staining samples were detected (Fig. 5D). The qPCR data of Raman (Fig. 5B) and staining samples (Fig. 5B) show the similar expression profiles. These results indicate that Raman measurement is highly comparable with conventional methods.

## Discussion

The conventional biochemical methods such as immunostaining and qPCR are widely used to evaluate the maturation level of MSC-osteoblast differentiation. However, most of biochemical methods are time-consuming, require a large amount of cells and cell fixation or lysed are needed. And the significance of histological staging is limited by inter-observer variability, in addition to the fact that the majority of mature bone cells fall into the intermediate range which is not easy to assess quantitatively. In this study, we propose and demonstrate that Raman spectroscopy can be a viable real-time, quantitative, in situ single cell biodetector for tissue engineering applications. Our results indicated that the Raman spectroscopy technique has higher sensitivity, is easier to quantitatively measure the mineralization level of cells and is more efficient than the conventional methods. Most importantly, detection via Raman spectroscopy can potentially achieved even with a single cell.

Cell morphology, gene expression of bone-related markers and staining assays of mineralization were examined to confirm the maturation of MSCs-derived osteoblasts, and we also compared those results by Raman spectroscopy. Figure 5 suggested the progression of MSCs into a more differentiated stage as mature osteoblasts as a successful osteogenic induction from MSCs.

Many of external environmental factors affect Raman signals such as the gamma rays, the dark current resulting from the thermal noise of CCD, fluorescent substances and unsuitable substrates. To evaluate and improve the stability of our Raman platform, we tested the Raman spectra of coverglass and quartz (Figure S1). Raman spectrum of coverglass has much more background noises with broad band peaks compared with the result of quartz which affects the measurement of Raman signal in MSCs. Therefore, we used quartz substrate due to its simple crystal property which provides a higher resolution of Raman measurement for cellular samples.

Raman signals of subtracts such as CH_2_ wag [64], Amide I [65] and Phe [66] have been used to normalize the intensity of Raman spectrum. Phe was considered for normalization, but the signal is not consistent during MSC-osteoblast differentiation due to the contribution of Phe in non-collagenous matrix protein. The signal of Amide I at 1500 to 1800 cm^−1^ shows a broadband in Raman spectrum which also affects the data normalization. All spectra were normalized by CH_2_ wag signal at 1449 cm^−1^ due to the high stability in MSCs during osteogenic induction. The Raman signals of Phe, CH_2_ wag and amide I were marked in yellow (Fig. 3A).

To confirm the signal intensity of mineral species, we increased integration time to enhance Raman signals. In addition, increasing the period of experimental operation cause cell death, the recorded Raman shift was cut down to 800–1500 cm^−1^. The enhanced signals of Raman spectra (Fig. 4A) are further improved by increasing the interrogation time compared with the results show in figure 3A.

Our results also indicate that OCP expresses in non-differentiated MSCs and significantly decreases after oeteogenic induction in MSCs. These suggest that OCP plays an essential role at early stage of mineralization during MSC-osteoblast differentiation. As the result of that, mineral-to-matrix ratio is very low in undifferentiated MSCs at day0 and day3 and significantly increases after day9.

On the other hand, β-TCP and collagen composite involved in the process of HAP synthesis as an enhancer which can be absorbed and further promote new bone formation and regeneration both *in vitro* and *in vivo*.[53], [67] Our results of demonstrated that β-TCP signal transient increase at Day 9 which may highly related to the expression of type I collagen (Fig. 5B and D).

However, the signals of HAP precursors, ACP and DCPD, did not show in our Raman spectra (Fig. 3A and 4A). We suggest that might because the productions of ACP and DCPD are too low in cells to detect during osteogenic differentiation in MSCs.

In this study, we developed a method using Raman spectroscopy to evaluate the maturation level of MSCs osteoblasts differentiation by monitoring level of mineral matrix. Raman spectra including HAP at 960 cm^−1^, OCP at 957 cm^−1^, and β-TCP at 970 cm^−1^ were slightly different at different stages of osteogenic differentiation.

In summary, we demonstrated that Raman spectroscopy is an excellent biosensor to detect the extent of maturation during MSCs-osteoblast differentiation in a non-disruptive, real-time and label free manner. Raman spectroscopy also provides objective data at the molecular level for not only stem cell research but also for the fields of biomedicine, especially tissue regenerative medicine in the near future.

### Conclusion

Although this technical platform cannot provide enough subcellular information in MSCs during oasteogenesis, base on our achievements we can introduce more powerful optical techniques such as surface-enhanced Raman scattering (SERS) and coherent anti-Stokes Raman scattering (CARS) into fields of biomedical research to further complement the deficits in traditional biological research. In this study, we established a unique detection method by using Raman spectroscopy to evaluate osteogenic differentiation of MSCs. The Raman-based platform enables rapid, real-time and sequential evaluation in a non cell-disruptive and label free manner. This platform technology provides accurate evaluation of osteogenic maturation in a small amount of sample; it can also make possible the further investigation the mechanism of mineral deposition during bone formation. Altogether, Raman spectroscopy may facilitate future advances in bone cell biology.

## Supporting Information

Figure S1 Raman spectra of coverglass and quartz for substrate.
(TIF)
