# Supplementary figures and images for: Detection of Osteogenic Differentiation by Differential Mineralized Matrix Production in Mesenchymal Stromal Cells by Raman Spectroscopy
Source: PLoS One. 2013 May 29;8(5):e65438. doi: 10.1371/journal.pone.0065438 (PMC3667172; doi:10.1371/journal.pone.0065438)

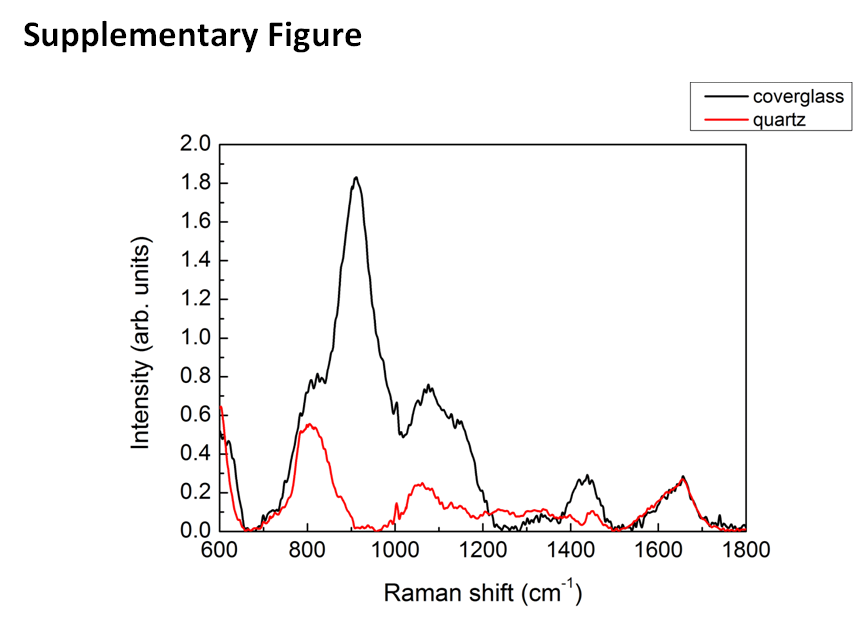

Supplement: Figure S1 — Raman spectra of coverglass and quartz for substrate. (TIF) [file pone.0065438.s001.tif]
